# Antioxidant, antimicrobial, and antiproliferative activity-based comparative study of peel and flesh polyphenols from *Actinidia chinensis*

**DOI:** 10.29219/fnr.v63.1577

**Published:** 2019-04-26

**Authors:** Aamina Alim, Ting Li, Tanzeela Nisar, Daoyuan Ren, Xichuan Zhai, Yaxing Pang, Xingbin Yang

**Affiliations:** Shaanxi Engineering Laboratory for Food Green Processing and Safety Control, College of Food Engineering and Nutritional Science, Shaanxi Normal University, Xi’an, China

**Keywords:** kiwifruit peel, kiwifruit flesh, polyphenols, antioxidant, antimicrobial, antiproliferative activity

## Abstract

**Background:**

Kiwifruit (*Actinidia chinensis*) peel has been always considered as useless because of the harsh taste. To promote the full utilization of kiwifruit resources it is essential to explore the nutritional benefits of kiwifruit peel.

**Objective:**

Our studies explored the difference in polyphenolic composition and biological activity including antioxidant, antimicrobial, and antiproliferative activity of the flesh and peel of kiwifruit.

**Design:**

Antioxidant activity of the extracted polyphenols of the peel and flesh of *A. chinensis* was checked by 2,2-diphenyl-1-picrylhydrazyl, 2,2’-azino-bis3-ethylbenzothiazoline-6-sulphonic acid (ABTS), hydroxyl ion reduction, and ion chelating ability. Antibacterial activity against *Escherichia coli*, *Listeria monocytogenes*, and *Staphylococcus aureus* and antiproliferative activity *against* HepG2 was tested in a dose- and time-dependent manner. Liquid chromatography/mass spectrometry (LC/MS) chromatogram of the peel and flesh further differentiated the phenolic acid profile.

**Results:**

The pericarp of kiwifruit was found to be more abundant in polyphenols and flavonoids than the flesh, with contents of 12.8 mg/g and 2.7 mg/g, respectively. LC/MS analysis revealed that the catachin, quercetin and epigallocatechin content (the main polyphenols in kiwifruit) in the peel was significantly higher than in the flesh (*P* < 0.05). The antioxidant and antibacterial activity of the peel was significantly higher when compared to the flesh. Moreover, the proliferation of HepG2 cells was time- and dose-dependently inhibited by kiwifruit polyphenols, with IC_50_ values of 170 μg/mL and 291 μg/mL for peel and flesh polyphenols after 72 h of treatment time, respectively.

**Conclusion:**

Kiwifruit peel, with higher content of phenolics and flavonoids, exerts more potent antioxidant, antibacterial, and anticancer activity than the flesh. Our study provides scientific evidence for the development of kiwifruit, especially peel-based, novel natural products with excellent bioactivity.

## Popular scientific summary

The pericarp of *Actinidia chinensis* exerts more potent antioxidant, antimicrobial and antiproliferative activity than the flesh because of significantly higher amount of phenolics and flavonoids.

Postharvest commodities, especially fruits and vegetables, have been found to contain a significant amount of active elements with several known biological and physiological benefits to human health. The kiwifruit is an economically important fruit crop because of its remarkably higher content of vitamin C and balanced nutritional composition of dietary fiber, minerals, and other health-beneficial metabolites (1). The kiwifruit is native to the Yangtze River valley of northern China and Zhejiang Province on the shore of eastern China (2). Annually, 1.06 million tons of kiwifruit is produced in China, which makes it the highest producer of kiwifruit in the world (3). At present, there are more than 70 known species around the world, but among them only *Actinidia chinensis* (golden kiwifruit) and *A. deliciosa* (green kiwifruit) are processed commercially (4). *Actinidia chinensis* cultivars are now being grown in many countries including China and have attracted much research interest during the past decade (5).

Studies to date concerning *A. chinensis* have mainly focused on its genome profiling and factors affecting the quality, bioactive constituents, and bioactivities of the fruit flesh (6–8). Extensive studies have reported the isolation and characterization of phenolic components of kiwifruit flesh by thin-layer chromatography, high performance liquid chromatography and ultra-high performance liquid chromatography (9, 10). Apart from the antioxidant activity, kiwifruit has also shown antimicrobial activity against many pathogenic bacteria: *Pseudomonas aeruginosa*, *Escherichia coli*, *Listeria monocytogenes*, and *Staphylococcus aureus* (11). Kiwifruit flesh has been under research for decades to explore its health benefits. Notably, kiwifruit peel is widely used for alcohol and citric acid production by solid state fermentation, suggesting its commercial potential. Indeed, many kinds of fruit peels are abundant in phenolic compounds and possess more effective biological action than the corresponding flesh (12). However, there are only two papers reporting on the polyphenol profile of the peel of kiwifruit (13, 14). Up until now, no study has illustrated the differences in phenolic profile and bioactivity between the peel and flesh of *A. chinensis*.

Therefore, this study was designed to investigate and compare the characteristics and biological functions of polyphenols present in *A. chinensis* peel and flesh. Specifically, the polyphenol and flavonoid contents were firstly investigated for the flesh and pericarp of *A. chinensis*. The antioxidant and antihepatoma properties of peel and flesh polyphenols were compared. The antimicrobial activity of peel and flesh polyphenols against *E. coli*, *L. monocytogenes*, and *S. aureus* was also evaluated by disk diffusion assay. Additionally, the specific phenolic profile of peel and flesh from *A. chinensis* was analyzed by Liquid chromatography/mass spectrometry (LC/MS).

## Materials and methods

### Reagents

Ethylenediaminetetraacetic acid (EDTA), metaphosphoric acid, sodium hydroxide, sodium nitrate, sodium carbonate aluminum nitrate, Folin–Ciocalteu reagent, 2,2-diphenyl-1-picrylhydrazyl (DPPH), hydrogen peroxide, salicylic acid, ferrous sulfate, ferrozine, ferrous chloride, formic acid, fetal bovine albumin, Roswell Park Memorial Institute medium (RPMI 1640), penicillin, streptomycin, phosphate buffer solution, and dimethyl sulfoxide (DMSO) were all procured from Sigma-Aldrich (Xian, China). High performance liquid chromatography grade acetonitrile, absolute methanol, and ethanol were purchased from Chemical Reagents Company (Xian, China). Ultra-pure water was created by a Milli-Q water-purification system (Millipore, Bedford, MA, USA). Bacterial strains (*E. coli* O157:H7 ATCC 700728, *S. aureus* ATTC 25923, and *L. monocytogenes* ATCC 35152) were collected from the College of Food Engineering and Nutritional Science, Shaanxi Normal University, Xi’an, China.

### Sample preparation and extraction of polyphenols

*Actinidia chinensis* was harvested at its commercial maturity level with 6.2 brix. All the fruits were washed with tap water and peeled. The peel and flesh were dried, weighed, and homogenized with a high-speed professional blender (Silex, Hamilton Beach, Virginia, USA) separately under liquid nitrogen for 1 min. Then all the samples were kept in a freeze dryer for 48 h (Virtis model 10-324, Midland, Canada). Finally, freeze-dried samples were ground to pass through 0.5 mm mesh, weighed, and kept at –20°C for further analysis (15).

Five grams of lyophilized peel and flesh samples were separately extracted with absolute methanol (1:20) using a stirring water bath (300 rpm) at 37°C. After 1 h the samples were filtered through Whatman no. 2 filter paper (Whatman International Limited, Kent, UK) under vacuum filtration. The residues were then extracted again with 80% (v/v) methanol (16). The two supernatants obtained were combined and concentrated using the rotatory evaporator (Eyela, Tokyo, Japan) at 37°C under low pressure. These concentrated polyphenols of flesh and peel were then placed in the freeze dryer for drying (17).

### Determination of total phenolic and total flavonoid contents

Total phenolic content (TPC) was determined by the Folin–Ciocalteu method as described by Jayaprakasha et al. (18) with slight modification. Twenty-five milligrams of polyphenols of flesh and peel were mixed with 10 mL of 60% ethanol (v/v) thoroughly. Then 2.4 mL of 0.2 N Folin–Ciocalteu reagent was added into 1 mL prepared sample and kept for 1 min. Each sample was mixed with 4.8 mL of 12% sodium carbonate prepared with water and supplemented with 60% ethanol for 10 mL total volume. Absorbance of samples and standards was checked at 760 nm after being kept in the dark for 2 h. Total phenolic content was calculated from the standard curve using gallic acid as standard (1–8 mg/mL).

Total flavonoid contents (TFC) of the flesh and peel were measured following the previously described method with slight modification (19). For sample preparation, 25 mg of lyophilized phenolic extracts of flesh and peel were thoroughly mixed with 10 mL of 60% ethanol (v/v). Then 2 mL of prepared sample was mixed with 0.4 mL of 5% sodium nitrite and kept for 6 min. Samples were then added with 0.4 mL 10% aluminum nitrate and kept for 6 min. Finally, 4 mL of 4% sodium hydroxide was added to each sample and kept for another 15 min. Absorbance of both samples and standards was recorded at a wavelength of 510 nm. Total flavonoid content was estimated from the standard curve using rutin as standard (1–8 mg/mL).

### Assessment of antioxidant activity

#### DPPH radical scavenging capacity

The DPPH free radical scavenging capacity was determined by following the procedure of Brandwilliams, Cuvelier, and Berset (20) with slight modifications. Briefly, 1 mL of flesh and peel extracts prepared in ethanol were mixed with 0.1 mM 3 mL of ethanol DPPH solution and kept in the dark for 30 min. The absorbance was determined at 517 nm to estimate the concentration of DPPH. Results were expressed as vitamin C equivalent antioxidant capacity with the following formula:

$$\text{Scavenging activity}\left(\text{\%}\right)=\left(1-\frac{{\text{A}}_{\text{sample}}}{{\text{A}}_{\text{Control}}}\right)\times 100$$ (1)

#### Radical-scavenging activity

2,2’-azino-bis3-ethylbenzothiazoline-6-sulphonic acid (ABTS) radical-scavenging activity was evaluated with the method described by Re et al. (21) with slight modification. ABTS radical was formed by mixing 7.4 mM ABTS solution with 2.6 mM potassium persulfate. The mixture was then allowed to stand for 12–16 h at room temperature in a dark place. The prepared solution was diluted with ethanol to an absorbance of 0.6~0.8 at 734 nm. One milliliter of flesh polyphenol, peel polyphenol, and vitamin C standards with different concentrations were mixed with 2 mL diluted ABTS radical. After 6 min of reaction time, the absorbance was checked at 734 nm. Results were expressed as vitamin C equivalent antioxidant capacity with the following formula:

$$\text{Scavenging activity}\left(\text{\%}\right)=\left(1-\frac{{\text{A}}_{\text{sample}}}{{\text{A}}_{\text{control}}}\right)\times 100$$ (2)

#### Hydroxyl ion reducing ability

The foraging activity of extracts on HO^•^ was determined by a modified Fenton-type reaction (22). Briefly, 1.0 mL of 6 mM FeSO_4_ was added into 1.0 mL extract of different concentrations (20–100 μg/mL). Each sample was supplemented with 1.0 mL of 6 mM H2O2 and 1.0 mL of 6 mM salicylic acid. After 30 min of reaction time, the absorbance was determined at 510 nm. The scavenging activity of flesh and peel extracts was calculated according to the following equation:

$$\text{Scavenging activity}\left(\text{\%}\right)=\left(1-\frac{{\text{A}}_{\text{sample}}}{{\text{A}}_{\text{control}}}\right)\times 100$$ (3)

#### Iron chelating capacity

The capacity of extracts to chelate Fe^2+^ was evaluated with the previous method (17). Thus 400 μL of each sample at different concentrations (20–100 μg/mL) was supplemented with 1,800 μL of methanol and 200 μL of 0.5 mM aqueous FeCl_2_. The control (EDTA) had all the reagents, excluding the samples. After 5 min, the reaction was initiated with the addition of 800 μL of 5.0 mM ferrozine, followed by 10 min of rest time. Finally, the absorbance at 562 nm was observed. The ferrous ion scavenging activity was calculated by the following equation:

$$\text{Chelating ability}\left(\text{\%}\right)=\left(1-\frac{{\text{A}}_{\text{sample}}}{{\text{A}}_{\text{control}}}\right)\times 100$$ (4)

### Antimicrobial activity

Tested microorganisms included gram-negative *E. coli* and gram-positive *S. aureus* and *L. monocytogenes*, which were cultured in Luria Bertani agar, tryptic soy agar, and nutrient agar, respectively. Antimicrobial activity of peel and flesh polyphenols at different concentrations (100, 200, and 300 μg/mL) was determined by the disk diffusion method. Each petri dish with 12 mL of sterile media was left for 1 h under sterile conditions for solidification and then evenly inoculated with 100 μL of 10^6^–10^7^ Colony forming unit (CFU)/mL inoculum for another 1 h. Then 10-mm sterilized disks were dipped in the prepared polyphenol extracts, placed in the middle of each inoculated petri plate, and incubated for 18–24 h at 37°C. A sterile disk not dipped into the polyphenol extract was used as control. All the tests for different concentrations and bacteria were done in triplicate (23).

### Cell culture

Human liver cancer HepG2 cells were obtained from the Cell Bank of Institute of Biochemistry and Cell Biology, Shaanxi Normal University (Xian, China). HepG2 cells were cultured in RPMI 1640 medium supplemented with 10% heat-inactivated fetal bovine serum and 1% of penicillin, streptomycin at 5% CO_2_, 37°C in 95% humidified air.

### Cell proliferation of HepG2

Colorimetric 3-(4,5-Dimethylthiazol-2-yl)-2,5- Diphenyltetrazolium Bromide (MTT) assay was performed to check cell viability. Cells (2×10^5^) seeded in 96-well plates were treated with 100 μL of peel and flesh polyphenols at various concentrations (100, 200, 300, and 400 μg/mL). After incubating for 24, 48, and 72 h, prepared MTT (10 μL) solution was added to each well and incubated for the next 2 h. Finally, each well was washed and DMSO was added to dissolve the formazan crystals that had formed. The absorbance was recorded at 570 nm and compared to the control, which did not contain polyphenols. The blank had no cells and polyphenols. Each sample was tested three times to define the cell proliferation rate (24). Cell proliferation (%) was calculated by the following formula:

$$\text{Cell proliferation}\left(\text{\%}\right)=\left(\frac{{\text{A}}_{\text{sample}}}{{\text{A}}_{\text{control}}}\frac{-{\text{A}}_{\text{Blank}}}{-{\text{A}}_{\text{Blank}}}\right)\times 100$$ (5)

### Identification and quantification of phenolic compounds with LC/MS

Freeze-dried polyphenols of peel and flesh were prepared in methanol and filtered through 0.22 μm Polyvinylidene fluoride (PVDF) membrane for LC/MS analysis. The identification of peel and flesh polyphenols was performed by LC/MS using electric spray ionization interface (1260-6460 triple Quad LC/MS, Agilent Technologies Inc., Massachusetts, USA). Vaporization heater and capillary heater were maintained at 100°C and 300°C, respectively. The analysis was carried out by following the methoof Lu et al. (25) with slight modifications. Briefly, analysis was conducted in the scan mode (m/z) from 100 to 1,000 in selected ion monitoring mode of negative (m/z) 464 for isoquercetin, (m/z) 306 for epigallocatechin, (m/z) 168 for dihydroxyphenylacetic acid, (m/z) 290 for catechin, (m/z) 194 for ferulic acid, (m/z) 288 for epicatechin, (m/z) 169 for gallic acid, (m/z) 286 for kaempferol, (m/z) 301 for quercetin and (m/z) 609 for rutin. The system was equipped with a 2.7 μm C_18_ column (AcclaimTMRSLC, 3.0 × 100 mm, Thermo Co. Ltd., Milford, MA, USA) and set at 17.20°C. Nitrogen was used as a nebulizer with a flow rate of 5.1 mL, while the collision gas was 11.01 mL/min.

Solvent A was 0.1% formic acid, and solvent B was acetonitrile. The flow rate was 0.2 mL/min. The mobile phase was programmed as follows: 0–20% B (v/v) from 0 to 4 min, 20–35% B (v/v) from 4 to 12 min, 35–65% B (v/v) from 12 to 18 min, 65–90% B (v/v) from 18 to 24 min, 90–5% B (v/v) from 24 to 32 min.

### Statistical analysis

The experimental data were analyzed by one-way analysis of variance using SPSS 13.0 (SPSS Inc., Chicago, USA) and expressed as means ± standard deviation (*n* = 3). The significant differences between mean values were calculated by Duncan’s multiple test at a significance level of *P* < 0.05.

## Results and discussion

### Total phenolic and flavonoid contents in peel and flesh of kiwifruit

**Fig. 1:**
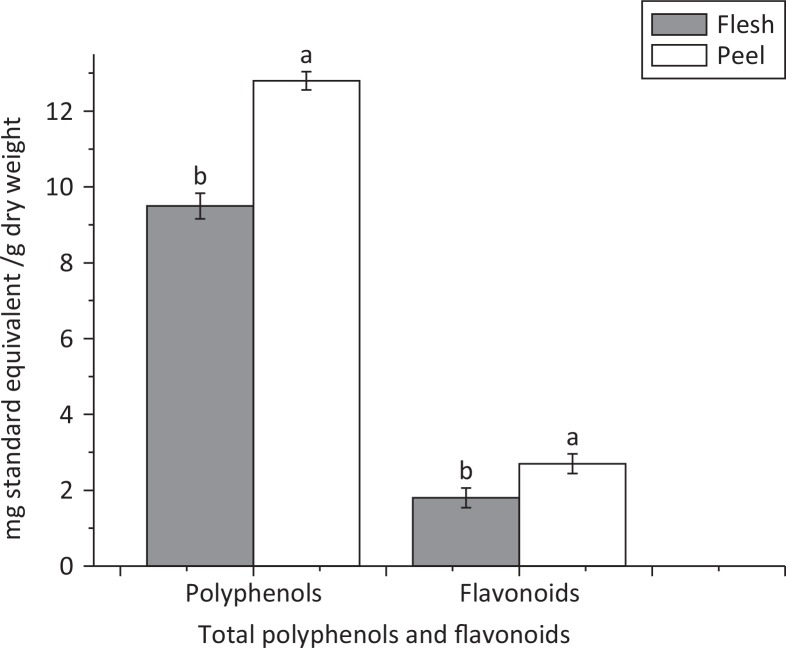
Total polyphenols and flavonoids in peel and flesh of *Actinidia chinensis*. Values are given as mean ± standard deviation. Different letters indicate significant difference (*P* < 0.05) analyzed by Duncan’s new multiple range test.

Phenolic components have been proven to act as reducing and antioxidant agents. Plant source phenols could inhibit the formation of superoxide-anion radicals formed by many enzymes (23, 26). Here, we measured the TPC and TFC of the extracts of flesh and peel from *A. chinensis* as shown in Fig. 1. The TPC determined by the Folin–Ciocalteu method was 9.5 and 12.8 mg gallic acid equivalent/g dry weight for the flesh and peel, respectively. Total phenolic content was estimated from the linear regression equation where the R^2^ was 0.992.

Flavonoids are the most well-known group of polyphenols present in the human diet. They are abundantly present in plant source food with many known health benefits. Quercetin is one of the known flavonoids in kiwifruit with anti-inflammatory, antibacterial, antiviral, and antioxidant activities (27, 28). Furthermore, flavonoids are proficient at chelating Cu^2+^, Fe^3+^, and Fe^2+^ cations. Complexes of flavonoids have been proven to be more effective superoxide radical scavengers than uncomplex flavonoids (29). Figure 1 shows the amount of flavonoids present in the flesh and peel of kiwifruit. The TFC (2.7 mg rutin/g) in peel was much higher (*P* < 0.05) than in flesh (1.8 mg rutin/g). The TFC was calculated by the linear regression equation where R^2^ = 0.9821. Our results are in compliance with previous studies (17, 30).

### Radical scavenging activity of kiwifruit peel and flesh towards DPPH and ABTS+

Many diseases like Alzheimer’s disease, reperfusion injury, inflammation, atherosclerosis, and Parkinson’s disease are linked to ROS-mediated Reactive oxygen species (ROS) damage to macromolecules because of the imbalanced radical scavenging and radical generating system (26). ABTS+ and 2,2-diphenyl-1-picrylhydrazyl (DPPH) assays are extensively used to estimate the radical scavenging activity of samples (31). In our studies, we also used ABTS+ and DPPH assays to evaluate the radical scavenging activity of peel and flesh polyphenols of *A. chinensis*.

**Fig. 2:**
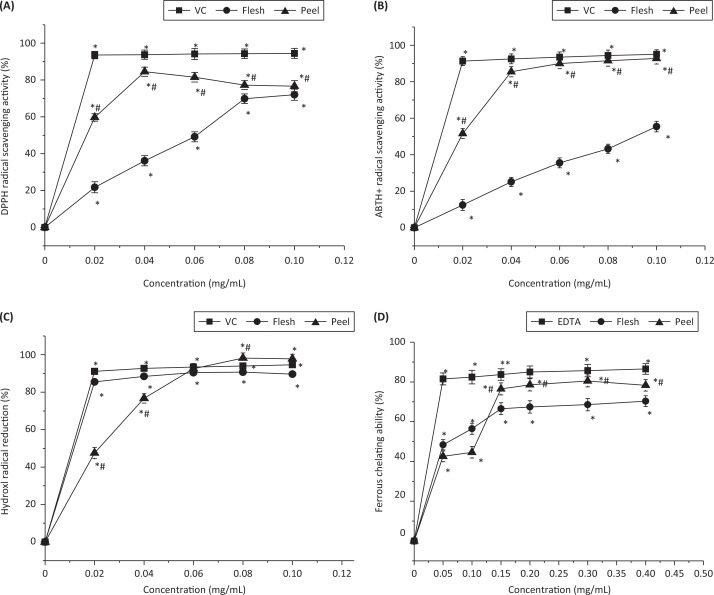
(a) DPPH radical scavenging activity, (b) ABTS+ radical scavenging activity, (c) hydroxyl radical reducing ability, and (d) ferrous chelating ability of peel and flesh polyphenols from *Actinidia chinensis*. VC: vitamin C. EDTA: ethylenediaminetetraacetic acid. ABTS+: 2,2’-azino-bis(3-ethylbenzothiazoline-6-sulphonic acid). DPPH: 2,2-diphenyl-1-picrylhydrazyl. Values are given as mean± standard deviation. *P < 0.05, treatments with different concentrations versus vehicle control treatment. #*P* < 0.05, peel polyphenols versus flesh polyphenols at the same concentration.

The free radical inhibition of kiwifruit flesh, peel, and standard antioxidant (vitamin C) at the concentration of 20 μg/mL was reduced by 21.8%, 59.7%, and 93.6%, respectively. As shown in Fig. 2a, the DPPH scavenging activity for the flesh at concentrations of 20, 40, 60, 80, and 100 μg/mL was 21.7%, 36.1%, 49.1%, 69.8%, and 72.0%, respectively, which gradually increased with the concentration. Similarly, the DPPH radical scavenging activity for the peel at concentrations of 20, 40, 60, 80, and 100 μg/mL was 59.7%, 84.5%, 81.5%, 77.2%, and 76.6%, respectively. These results show that both kiwifruit flesh and peel have good radical scavenging activity, but the polyphenols of peel exerted much more effective free radical scavenging activity (*P* < 0.05). However, the radical scavenging activity of kiwifruit flesh and peel are both lower than that of vitamin C (Fig. 2a).

An additional effective technique to quantify radical scavenging activity is the ABTS+ radical cation decolorization assay, which can present comparable results to those attained in the DPPH assay. Figure 2b expresses the changes in the radical scavenging activity of both kiwifruit peel and flesh towards ABTS radicals. The ABTS+ scavenging activity of flesh at 20, 40, 60, 80, and 100 μg/mL was 12.4%, 25.1%, 35.5%, 43.2%, and 55.4%, respectively. Gradual increase can be observed with increased concentration of kiwifruit flesh. Similarly, a gradual increase in the radical scavenging activity of the peel can be seen at 20, 40, and 60 μg/mL, which is 51.6%, 85.5%, and 90.0%, respectively. A further increase in concentration did not bring any significant changes in the values. Accordingly, kiwifruit peel extract was found to have more potent radical scavenging activity than flesh extract.

### Hydroxyl radical reducing ability of kiwifruit

Amongst the many oxygen radicals, HO^•^ is the most active one and can bring oxidative damage to many biomolecules, thereby causing aging, cancer, and numerous other diseases (17). Figure 2c explains the dose-dependent curve of the HO^•^-scavenging activities of the flesh and peel of kiwifruit. At a concentration of 80 μg/mL, peel polyphenols exerted the highest HO^•^-scavenging activity (98.2%), while the values for flesh and vitamin C were 90.7% and 93.9%, respectively. However, vitamin C at concentrations of 20 μg/mL (91.14%) and 40 μg/mL (92.69%) expressed higher activity, as compared to the flesh and peel, respectively. The HO^•^-scavenging activity of flesh at concentrations of 20, 40, 60, 80, and 100 μg/mL was 85.4%, 88.4%, 90.4%, 90.7%, and 89.7%, respectively. By contrast, the activity of peel polyphenol increased gradually from 47.5 to 97.8% with an increase in concentration (20–100 μg/mL). The peel polyphenols at high concentrations were found to have higher hydroxyl radical scavenging activity than the positive control because of the presence of polyphenols, which act by giving the electron and react with the free radical, further converting these radicals to a stable form (33).

### Iron chelating activity of kiwifruit

Ferrozine has the capability to make complexes with iron (Fe^2+^). In the presence of chelating agents, these complexes will dislocate and cause a decrease in the red color of the solution. Figure 2d represents the iron chelating activity of kiwifruit flesh and peel polyphenols. At the initial concentration (50 μg/mL), peel polyphenols exhibited the lowest chelating activity (42.6%), as compared to the flesh polyphenols and EDTA, which were 48.4% and 81.5%, respectively. With a gradual increase in concentration, the metal chelating activity of both peel and flesh polyphenols increased gradually but was still lower than that of EDTA. When the concentration increased to 150 μg/mL, the chelating activity of peel polyphenols (76.5%) was higher than that of flesh polyphenols (66.6%). At a concentration of 300 μg/mL, peel polyphenols exhibited the maximum chelating capacity (80.5%) as compared to flesh polyphenols (68.6%). Overall, peel polyphenols exhibited significantly more potent metal chelating activity than flesh polyphenols (*P* < 0.05) but weaker than the positive control EDTA.

### Antimicrobial activity of kiwifruit

The antimicrobial activity of flesh and peel polyphenols against *E. coli*, *S. aureus*, and *L. monocytogenes* is presented in Table 1. Both peel and flesh polyphenols dose-dependently inhibited the growth of the three bacterial strains. At concentrations of 100 μg/mL, 200 μg/mL, and 300 μg/mL, peel polyphenols exhibited the most effective antibacterial activity towards gram-positive *S. aureus*, which was 13.7 ± 0.4 mm, 16.2 ± 0.6 mm, and 20.4 ± 0.3 mm, respectively, as compared to other tested bacteria. For flesh polyphenols, the activity at 100, 200, and 300 μg/mL against *S. aureus* was 11.4 ± 0.3 mm, 12.6 ± 0.2 mm, and 14.7 ± 0.3 mm, respectively. Similarly, peel polyphenols at concentrations of 100, 200, and 300 μg/mL (13.1 ± 0.4 mm, 15.7 ± 0.2 mm, and 17.4 ± 0.6 mm) showed higher antibacterial activity towards *L. monocytogenes* than flesh polyphenols (10.7 ± 0.4 mm, 12.3 ± 0.5 mm, and 13.9 ± 0.4 mm). With regard to the gram-negative *E. coli*, peel polyphenols at 100, 200, and 300 μg/mL exerted 10.6 ± 0.6 mm, 13.4 ± 0.4 mm, and 15.9 ± 0.5 mm of antimicrobial activity, respectively. Flesh polyphenols at 100 μg/mL showed no antimicrobial activity against *E. coli*, while at 200 and 300 μg/mL, the activity was increased to 11.3 ± 0.3 mm and 12.4 ± 0.4 mm, respectively. Taken together, peel polyphenols were found to have significantly more potent antibacterial property against both gram-positive and gram-negative bacteria than flesh polyphenols (*P* < 0.05).

**Table 1:** Antimicrobial activity of flesh and peel polyphenols of *Actinidia chinensis*

Microorganisms		Flesh (μg/mL)			Peel (μg/mL)		
		100	200	300	100	200	300
**Gram negative**	*E. coli*	-	11.3±0.30b	12.4±0.40b	10.6±0.62a	13.4±0.47a	15.9±0.50a
**Gram positive**	*S. aureus*	11.4±0.34b	12.6±0.25b	14.7±0.37b	13.7±0.40a	16.2±0.62a	20.4±0.30a
	*L. monocytogenes*	10.7±0.42b	12.3±0.50b	13.9±0.41b	13.1±0.40a	15.7±0.20a	17.4±0.60a

*E. coli*: *Escherichia coli*, *S. aureus*: *Staphylococcus aureus*, *L. monocytogenes*: *Listeria monocytogenes*. Antimicrobial activity was assessed by measuring the inhibition zones in diameter of the paper disk (10 mm). –: no activity. Values are expressed as mean ± SD of triplicate assays. Different letters indicate significant differences (*P* < 0.05) of peel and flesh polyphenols at the same concentration and for the same bacteria.

Gram-positive microorganisms (*L. monocytogenes* and *S. aureus*) were more vulnerable to kiwifruit polyphenols than the gram-negative ones (*E. coli*), as evidenced by the larger inhibition zones for the gram-positive bacteria in response to polyphenols (Table 1). Gram-negative bacteria were found to have an outer hydrophilic membrane comprising lipopolysaccharide molecules that act as barrier to hydrophobic compounds. This barrier reduces but does not block the diffusion of hydrophobic compounds (34). These results are in accordance with the previous study (11), where the antimicrobial activity of kiwi extracts is more effective on *S. aureus* than *E. coli*.

### Effects of kiwifruit on proliferation of HepG2 cells

**Fig. 3:**
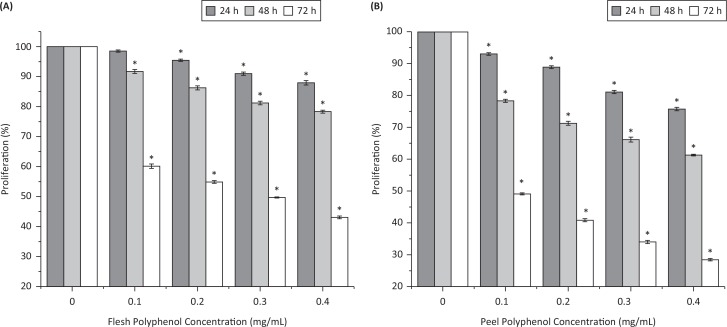
Inhibitory effects of (a) flesh polyphenols and (b) peel polyphenols from *Actinidia chinensis* against HepG2 cell proliferation (%) after 24, 48, and 72 h of treatment time. Values are given as mean ± standard deviation. *P < 0.05, treatments with different concentrations versus vehicle control treatment.

Figure 3 shows the effect of flesh and peel polyphenols at 100, 200, 300, and 400 μg/mL on the proliferation of human liver cancer HepG2 cells. The proliferation of HepG2 cells was inhibited by both peel and flesh polyphenols in dose- and time-dependent manners. After 24 h of treatment, cell viability declined to 87.9% for 400 μg/mL of flesh and to 75.7% for the same concentration of peel. After being treated for 48 h, cell viability decreased to 91.7, 86.2, 81.2, and 78.3% for flesh polyphenols and 78.3, 71.2, 66.1, and 61.2% for peel polyphenols at concentrations of 100, 200, 300, and 400 μg/mL, respectively. After being treated for 72 h, the inhibition rates of flesh polyphenols at 100–400 μg/mL were 39.8%, 45.1%, 50.3%, and 56.9%, respectively, while those of peel polyphenols were 50.9%, 59.1%, 66.0%, and 71.6%, respectively. The IC_50_ value against HepG2 cells was 170.0 μg/mL for peel polyphenols and 291.0 μg/mL for flesh polyphenols with 72 h of treatment time.

Robust research has revealed a strong relationship between phenolic compounds and anticancer activity (35). Our results indicated that kiwifruit peel polyphenols, containing higher content of phenolics and flavonoids, exerted more potent antihepatoma activity than flesh polyphenols. These results are comparable to previous results, where polyphenols extracted from the peel of apple and mango inhibited the growth of prostate cancer cells by 30% and HepG2 cells by 50%, respectively (36, 37). Similarly, hawthorn flesh and peel extracts showed IC_50_ values of 175.5 and 88.6 lg/mL against MCF-7 breast cancer cells (38). Our study provides new evidence for a positive correlation between polyphenols and antitumor efficacy.

### Phenolic profile of kiwifruit flesh and peel

**Fig. 4:**
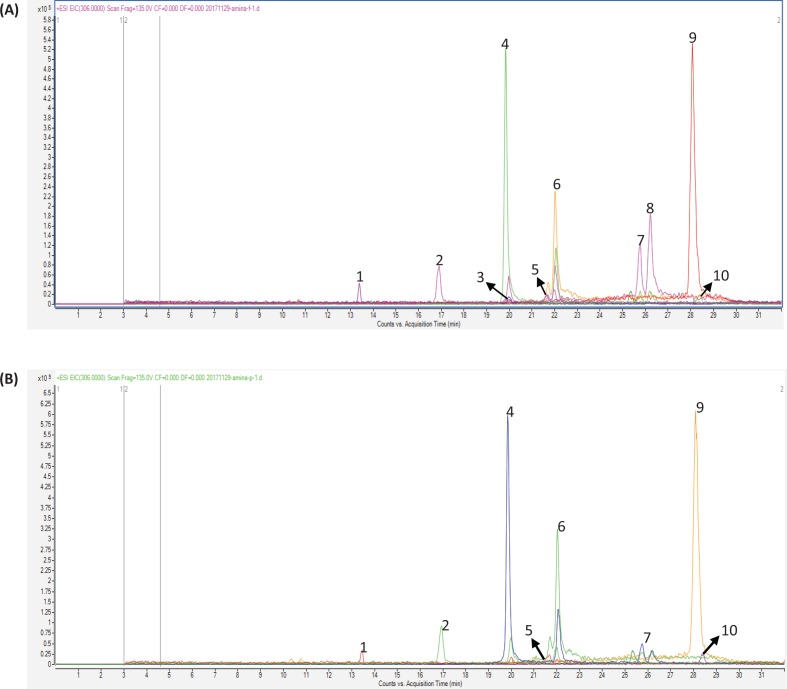
HPLC/MS chromatogram of (a) flesh polyphenols and (b) peel polyphenols from *Actinidia chinensis*.

To identify the differences in the specific phenolic profile of kiwifruit flesh and peel, LC/MS chromatography was conducted. Figure 4a and b, which shows clearly separated peaks, depicts the phenolic profiles of the kiwifruit peel and flesh, respectively. Table 2 further elaborates the retention time and area size of individual components. The major phenolic compounds in kiwifruit are catechin, epicatechin, quercetin, and epigallocatechin, with 29.34 ± 0.21%, 15.84 ± 0.28%, 44.88 ± 0.61%, and 5.29 ± 0.36% of area size for peel polyphenols, respectively, and with 23.95 ± 0.37%, 15.46 ± 0.43%, 36.41 ± 0.21%, and 4.64 ± 0.40% for flesh, respectively. In addition, the contents of isoquercetin (1.47 ± 0.51%) and gallic acid (1.80 ± 0.53%) in peel were slightly higher than that of flesh polyphenols. Prominently, kaempferol and dihydroxyphenyl lactic acid were not found in kiwifruit peel, whereas the contents of these in the flesh were 13.51 ± 0.25% and 0.82 ± 0.22%, respectively. With low content in kiwifruit, both ferulic acid and rutin were slightly more abundant in flesh than in peel. These results are comparable with a previous study where quercetin, catechins, epicatechins, and rutin were identified in kiwifruit peel extracts (14). Our study illustrated that *A. chinensis* peel has a higher amount of specific polyphenols and flavonoids, which may be responsible for the higher antioxidant, antimicrobial, and antiproliferative activity as compared to the flesh.

**Table 2:** Phenolic compounds identified by High performance liquid chromatography/mass spectrometry (HPLC/MS) in flesh and peel of *Actinidia chinensis*

Given number	Compound	Retention time	MS (m/z)	Flesh (area %)	Peel (area %)
1	Isoquercetin	13.38	464	1.46±0.32a	1.47±0.51a
2	Epigallocatechin	16.90	306	4.64±0.40b	5.29±0.36a
3	Dihydroxyphenylacetic acid	19.98	168	0.82±0.22a	N
4	Catechin	19.84	290	23.95±0.37b	29.34±0.21a
5	Ferulic acid	21.66	194	1.25±0.51a	0.66±0.41b
6	Epicatechin	22.02	288	15.46±0.43a	15.84±0.28a
7	Gallic acid	25.36	169	1.36±0.52a	1.80±0.53a
8	Kaempferol	26.21	286	13.51±0.25a	N
9	Quercetin	28.06	301	36.41±0.21b	44.88±0.61a
10	Rutin	28.36	609	1.13±0.36a	0.72±0.53a

m/z: mass to charge ratio, N: not found. Values are expressed as mean ± SD of triplicate assays. Different letters in the same column indicate significant differences (*P* < 0.05).

## Conclusion

In the present study, we demonstrate that the pericarps of *A. chinensis* exert much more potent antioxidant, antibacterial. and antihepatoma activities than the flesh. The chemical composition analysis shows that the peel of *A. chinensis* contains higher amounts of polyphenols and flavonoids than the flesh, with catechin, quercetin, and epigallocatechin showing significantly higher abundance in the pericarp. These findings indicate that the polyphenols, which exist more abundantly in kiwifruit peel, may be responsible for the remarkable bioactivity of kiwifruit. Our study provides scientific evidence for the development of kiwifruit-based novel natural products with potent antioxidant, antibacterial, and anticancer activities. In consideration of the abundant phenolics and outstanding bioactivities of kiwifruit peel, we recommend more extensive industrial usage of kiwifruit peel.
